# Development and validation of a prognostic model for patients with cT1-4N1-3M1 esophageal squamous cell carcinoma: based on the SEER database and the Chinese cohort study

**DOI:** 10.3389/fonc.2025.1547462

**Published:** 2025-04-28

**Authors:** Xiao-Mei Wang, Can-Tong Liu, Jia-Tao Huang, Zhi-Han Zhang, Yi-Wei Xu, Fang-Cai Wu, Yu-Hui Peng

**Affiliations:** ^1^ Department of Clinical Laboratory Medicine, The Cancer Hospital of Shantou University Medical College, Shantou, China; ^2^ Esophageal Cancer Prevention and Control Research Center, The Cancer Hospital of Shantou University Medical College, Shantou, China; ^3^ Guangdong Esophageal Cancer Research Institute, Guangzhou, China; ^4^ Department of Radiation Oncology, The Cancer Hospital of Shantou University Medical College, Shantou, China

**Keywords:** ESCC, SEER, overall survival, nomogram, cT1-4N1-3M1

## Abstract

**Objective:**

The purpose of this study was to investigate the impact of clinicopathological factors on the overall survival (OS) of advanced esophageal squamous cell carcinoma (ESCC) patients with both lymph node and distant metastasis and build a nomogram for OS prediction.

**Method:**

We selected 621 ESCC patients with cT1-4N1-3M1 stage without surgical treatment from the Surveillance, Epidemiology, and End Results (SEER) database and randomized (in a 7:3 ratio) to the training cohort and internal validation cohort. Another 159 patients were enrolled from the Cancer Hospital of Shantou University Medical College as the external validation cohort. A nomogram was developed based on independent risk factors that resulted from a multivariate Cox regression analysis. Receiver operating characteristic (ROC) curves and the area under the ROC curve (AUC) were used to evaluate the discriminative ability and calibration curves were constructed to evaluate the calibration ability. Kaplan-Meier survival analysis and log-rank tests were then used to predict the further OS status of these patients.

**Results:**

The multivariate Cox regression analysis revealed that sex, T stage, radiotherapy, and chemotherapy were independent prognostic factors for ESCC patients with cT1-4N1-3M1 stage. All these factors were incorporated to construct a nomogram. The prognostic nomogram in training cohort exhibited the AUCs of 0.784, 0.746, and 0.735 for predicting 6-, 9-, and 12-month OS, respectively. Calibration curves exhibited that the nomogram-predicted OS were insistent with the actual OS. In validation cohorts, the nomogram still showed acceptable discrimination ability and calibration. All individuals were allocated into high-risk versus low-risk groups based on the median risk score of the training cohort. The OS of the high-risk group was shorter than that of the low-risk group in three cohorts.

**Conclusion:**

We developed and validated an individualized survival prediction nomogram for predicting OS in ESCC patients with cT1-4N1-3M1 stage, which may help clinicians to assess the situation of advanced ESCC patients and implement further treatment.

## Introduction

According to the latest cancer statistics, esophageal cancer (EC) is the eleventh most commonly diagnosed cancer and the seventh leading cause of cancer death worldwide, with an estimated 511,000 new cases and 445,000 deaths in 2022 (1). Squamous cell carcinoma (SCC) is the predominant histological subtype in Asian countries, and covers more than 90 percents of all EC (2). The esophagus has a unique network of capillary lymphatic vessels, making the lymphatic drainage system extremely complex (3). Lymph node metastasis (LNM) is one of the most common methods of EC metastasis. About 52% of EC patients have LNM, and its LNM areas mainly include mediastinal lymph nodes, cervical lymph nodes, and abdominal lymph nodes (4). LNM is an important mechanism for the occurrence and development of EC, and an important factor for the poor prognosis (5). What’s worse, approximately half of patients have distant metastasis (DM) at initial diagnosis and more than one-third develop DM after surgery or radiotherapy. DM mostly develops within six months of radical treatment, and median survival after diagnosis of DM is only five months (6–8).

In the past ten years, with the progress of diagnostic and treatment technologies, both the incidence and mortality of EC have been decreasing. However, traditional curative esophagectomy and chemotherapy bring little benefit for patients with advanced EC, so in recent years, the medical community has been exploring new therapeutic strategies, such as targeted therapy, immunotherapy and multidisciplinary integrated treatment mode. In 2020, YNOTE-590 study (9) announced for the first time the results of pembrolizumab combined with chemotherapy in the first-line treatment of advanced EC, which reached the preset end points of progression-free survival (PFS) and overall survival (OS), and became the first approved first-line immunotherapy regimen for advanced EC. At the ASCO-GI Conference in 2024, KEYNOTE-590 study (10) showed that in metastatic ESCC patients, pembrolizumab combined with chemotherapy increased the 5-year OS rate compared with chemotherapy alone. Although first-line immunotherapy combined with chemotherapy resulted in improved response rates and prolonged OS for more than 1 year, only 15% of patients experienced long-term benefit. What’s more, patients with advanced EC with both LNM and DM (cT1-4N1-3M1) have a poorer prognosis (5, 11). In spite of this, there are still some “survival with tumor” patients who have long-term stability and tend to improve after systemic effective anti-tumor therapy (12). Therefore, predicting prognosis is in urgent need for seeking for patients with longer survival and developing personalized treatment strategies for them.

At present, the prognostic predictors of advanced EC include the characteristics of the primary tumor, tumor markers, genes expression, treatment options, and laboratory test indicators (13–15). Nomograms are graphical evaluation systems for visualizing the results of predictive models that can quantify risk based on statistical prediction models (16, 17). Many studies have shown that a nomogram can be used as a supplementation to the AJCC TNM staging system to individually predict patient prognosis and help physicians to select the best treatment for individual clinicopathological conditions (18–20). However, treatment options and survival in advanced EC differ significantly from those in the early stages. We aimed to investigate the impact of clinicopathological factors on the OS and build a prognostic nomogram for predicting the survival of advanced ESCC patients with both LNM and DM using the Surveillance, Epidemiology, and End Results (SEER) database and validated in a Chinese cohort.

## Materials and methods

### Patient datasets and research design

**Figure 1:**
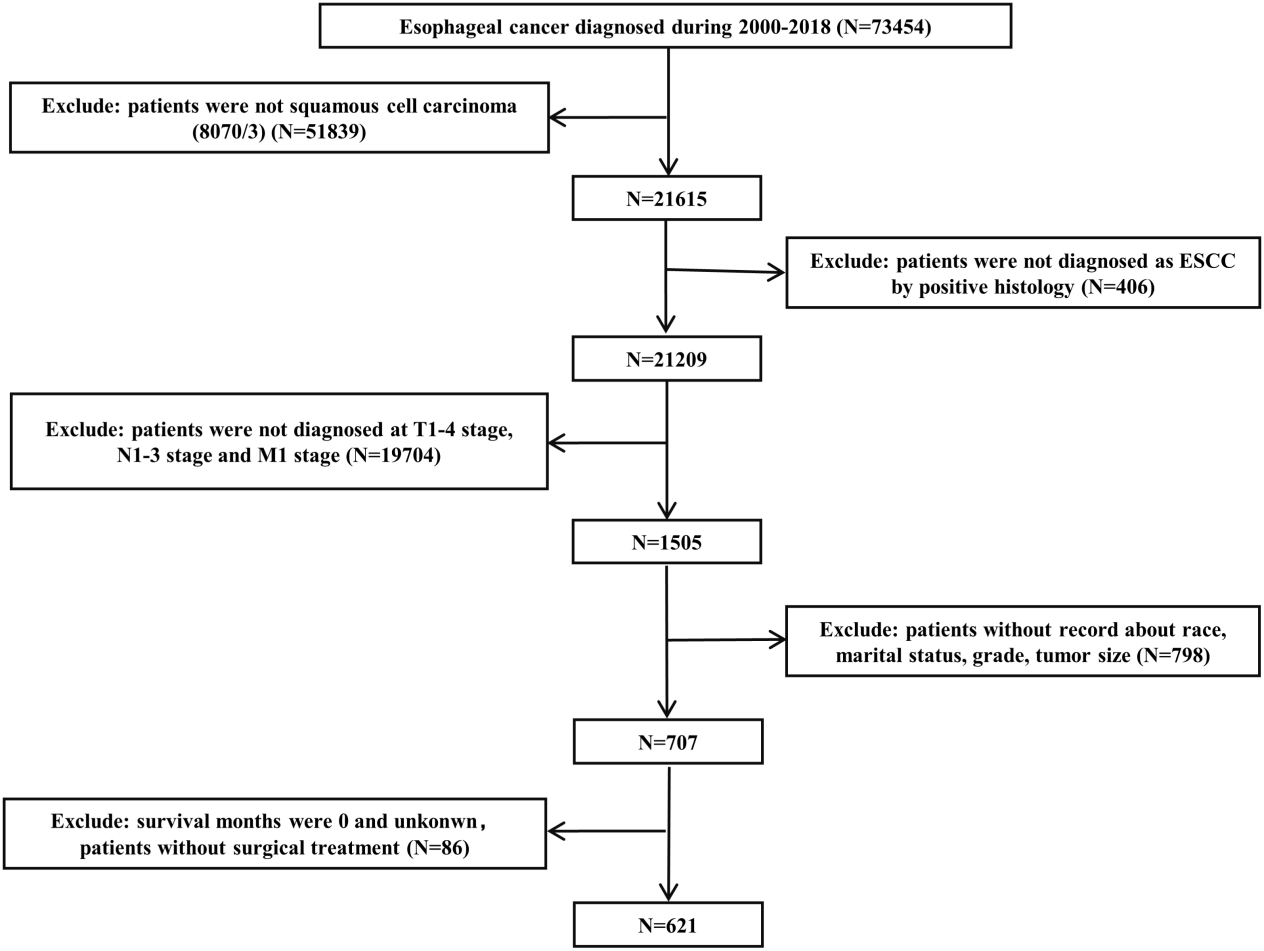
Flow chart of the study. ESCC, esophageal squamous cell carcinoma.

The SEER database is one of the most representative large tumor registration databases in the North America. Data were retrieved from the SEER*Stat software (version 8.4.2). We applied strict inclusion and exclusion criteria during the enrollment process. Patients diagnosed as primary ESCC by positive histology and classified as cT1-4N1-3M1 stage were included. The exclusion criteria were as follows: (1) patients who were not squamous cell carcinoma (8070/3); (2) patients without record detailed information, including race, marital status, grade, tumor size and survival months; and (3) patients who underwent surgery at the first admission. Total 621 ESCC patients diagnosed with cT1-4N1-3M1 stage were enrolled within the SEER database from 2000 to 2018. The flowchart of case selection is presented in **Figure 1**. The enrolled patients were randomized (in a 7:3 ratio) to a training cohort and an internal validation cohort. The external validation cohort consisted of ESCC patients with cT1-4N1-3M1 stage without surgical treatment who attended the Cancer Hospital of Shantou University Medical College from 2000-2022. Using the similar screening criteria, we eventually recruited 159 patients. All patients were restaged according to the AJCC eighth-edition staging principles.

### Variables declaration

Pathological and clinical information from the SEER database includes age, sex, race, marital status, primary site, grade, tumor size, TNM stage, radiotherapy, chemotherapy, survival months and vital status. For these variables, we subclassified the patients by age (< 60 and ≥ 60), race (White, Black, and others), marital status (married, unmarried, and others), grade (Grade I-II and Grade III-IV), radiation (no/other and yes), and chemotherapy (no/unknown and yes). The optimal cut-off value for tumor size was determined using X-tile software (Version 3.6.1, Yale University). The primary site was reclassified based on the International Classification of Diseases for Oncology (ICD-O-3) codes (upper: C15.0 and C15.3; middle: C15.4 and C15.1; lower: C15.2 and C15.5; others: overlapping lesions/NOS). In this study, the primary endpoint was OS, which was between the date of diagnosis and the date of death from any cause or the last follow-up visit.

### Nomogram construction and evaluation

Categorical variables were adopted for all continuous variables in three cohorts, according to the optimal cut-off values determined by the X-tile program. Cox proportional hazards regression analysis was performed using SPSS software to calculate the hazard ratio (HR) and 95% confidence interval (CI) for selected factors associated with OS. Variables with *P* values less than 0.05 were screened in the univariate Cox regression analysis, and then multivariate Cox proportional hazards regression analysis was applied to identify independent prognostic factors.

A nomogram was developed based on independent risk factors that resulted from the multivariate Cox regression analysis. Each independent prognostic factor in the nomogram was assigned a risk score, and the total risk score was calculated from the patient data to predict 6-, 9- and 12-month OS of ESCC patients with cT1-4N1-3M1 stage in the training cohort. To evaluate the discriminative ability, we constructed a receiver operating characteristic (ROC) curve. The nomogram discrimination efficacy was assessed by the area under the ROC curve (AUC). Calibration curves were constructed by using a bootstrap method for 1000 resamples to compare the compatibility of the nomogram-predicted OS and the actual OS. Kaplan-Meier survival analysis and log-rank tests were then used to predict the further OS status of these patients.

### Statistical analysis

In this study, statistical analysis was conducted using IBM SPSS Statistics (version 20.0, USA), X-tile software (version 3.6.1, Yale University) and R software (version 4.1.3, https://www.r-project.org/). X-tile was employed to determine optimal cut-off values for converting continuous variables into binary variables. Patients from the SEER database were randomly divided into a training cohort and an internal validation cohort in a 7:3 ratio using R software. Intergroup comparisons of patient characteristics were performed using chi-square tests or Fisher’s exact tests. Univariate Cox regression analysis was performed for clinicopathological characteristics in the training cohort. In order to control the influence of confounding variables, multivariate Cox regression analysis was performed for the factors with *P* < 0.05, and stepwise backward regression was used to further screen the variables. Based on independent prognostic factors obtained from Cox regression analysis, a prognostic nomogram was constructed using the *“rms”* package in R software to predict the 6-, 9-, and 12-month OS rates of ESCC patients with cT1-4N1-3M1 stage. The *“timeROC”* package was used to generate ROC curves and calculate AUC values for model performance evaluation. Calibration curves were plotted to assess model calibration accuracy. The calibration of the model was evaluated by plotting a calibration curve. Finally, Kaplan-Meier curves and log-rank tests were then used for survival analysis. A two­sided *P* < 0.05 was considered statistically significant for all analyses.

## Results

### Characteristics of participants

**Table 1** showed the clinicopathologic characteristics of ESCC patients with cT1-4N1-3M1 stage in the training and validation cohorts. 621 ESCC patients with cT1-4N1-3M1 stage were enrolled in the SEER database and randomized into a training cohort (n = 435) and an internal validation cohort (n = 186), with no differences in clinicopathological and demographic characteristics between both cohorts. The 621 patients from the SEER database were mostly elderly, and more males than females (74.1% vs. 25.9%). More than half of the patients were white and married. 259 (41.7%) patients had primary tumors located in the middle/thoracic of the esophagus, with a median tumor size of 5.1 cm. AJCC staging showed that most patients were in T4 (34.6%) or N1 (87.1%), and the distribution of patients in grade I-II and grade III were more evenly distributed with 281 (45.2%) and 340 (54.8%) patients, respectively. 64.9% patients received radiotherapy, and 71.0% received chemotherapy. All enrolled 159 ESCC patients in the external validation cohort were married Asians, and most of them were greater than 60 years old. All patients received chemotherapy, besides 97.5% patients receiving radiotherapy.

**Table 1:** Demographics and clinicopathologic characteristics of included patients.

Characteristics	Training cohort (n = 435)	Internal validation cohort (n=186)	Overall (n = 621)	External validation cohort (n=159)
Age				
<60	124 (28.5%)	67 (36.0%)	191 (30.6%)	74 (46.5%)
≥60	311 (71.5%)	119 (64.0%)	430 (69.2%)	85 (53.5%)
Sex				
Female	112 (25.7%)	49 (26.3%)	161 (25.9%)	49 (30.8%)
Male	323 (74.3%)	137 (73.7%)	460 (74.1%)	110 (69.2%)
Race				
White	269 (61.8%)	112 (60.2%)	381 (61.4%)	0 (0.0%)
Black	105 (24.1%)	59 (31.7%)	164 (26.4%)	0 (0.0%)
Others^a^	61 (14.0%)	15 (8.1%)	76 (12.2%)	159 (100.0%)
Marital status				
Married	230 (52.9%)	82 (44.1%)	312 (50.2%)	159 (100.0%)
Unmarried	90 (20.7%)	51 (27.4%)	141 (22.7%)	0 (0.0%)
Others^b^	115 (26.4%)	53 (28.5%)	168 (27.1%)	0 (0.0%)
Primary site				
Upper	48 (11.0%)	28 (15.1%)	76 (12.2%)	48 (30.2%)
Middle/Thoracic	182 (41.8%)	77 (41.4%)	259 (41.7%)	79 (49.7%)
Lower	138 (31.7%)	55 (29.6%)	193 (31.1%)	13 (8.2%)
Others^c^	67 (15.4%)	26 (14.0%)	93 (15.0%)	19 (11.9%)
Grade				
Grade I-II	185 (42.5%)	96 (51.6%)	281 (45.2%)	17 (10.7%)
Grade III	250 (57.5%)	90 (48.4%)	340 (54.8%)	15 (9.4%)
Tumor size (cm)				
≤ 6	278 (63.9%)	119 (64.0%)	397 (64.0%)	78 (49.1%)
> 6	157 (36.1%)	67 (36.0%)	224 (36.1%)	48 (30.2%)
T stage				
T1	134 (30.8%)	57 (30.6%)	191 (30.8%)	1 (6.3%)
T2	26 (6.0%)	10 (5.4%)	36 (5.8%)	16 (3.1%)
T3	125 (28.7%)	54 (29.0%)	179 (28.8%)	87 (54.7%)
T4	150 (34.5%)	65 (35.0%)	215 (34.6%)	55 (34.6%)
N stage				
N1	371 (85.3%)	170 (91.4%)	541 (87.1%)	121 (76.1%)
N2	46 (10.6%)	11 (6.0%)	57 (9.2%)	29 (18.2%)
N3	18 (4.1%)	5 (2.7%)	23 (3.7%)	9 (5.7%)
Radiotherapy				
No/Other	157 (36.1%)	61 (32.8%)	218 (35.1%)	4 (2.5%)
Yes	278 (63.9%)	125 (67.2%)	403 (64.9%)	155 (97.5%)
Chemotherapy				
No/Unknown	125 (28.7%)	55 (29.6%)	180 (29.0%)	0 (0.0%)
Yes	310 (71.3%)	131 (70.4%)	441 (71.0%)	159 (100.0%)

Others^a^, including Asian or Pacific Islander and American Indian/Alaska Native; Others^b^, including separated, divorced and widowed; Others^c^, including overlapping lesion of esophagus and not otherwise specified.

### Cox proportional hazards regression analysis for variables screening

In the training cohort, univariate Cox regression analysis demonstrated that sex, T stage, radiotherapy, and chemotherapy were significantly associated with OS (*P* < 0.05). After controlling for confounding variables with multivariate Cox regression, 4 variables were ultimately identified as independent prognostic variables, including sex (HR: 1.268; 95% CI: 1.016-1.582; *P* = 0.036), T3 stage (HR: 0.777; 95% CI: 0.604-0.999; *P* = 0.049), T4 stage (HR: 1.270; 95% CI: 1.000-1.614; *P* < 0.050), radiotherapy (HR: 0.740; 95% CI: 0.603-0.907; *P* = 0.004), chemotherapy (HR: 0.295; 95% CI: 0.234-0.373; *P* < 0.004). The outcomes of the Cox regression survival analysis based on OS are presented in **Table 2**.

**Table 2:** Selection of variables independently associated with OS by univariate and multivariate Cox proportional hazards analysis in the training cohort.

Characteristics	Univariate analysis		Multivariate analysis	
	HR (95% CI)	*P* value	HR (95% CI)	*P* value
Age				
<60	Ref.			
≥60	1.105 (0.894-1.367)	0.355		
Sex				
Female	Ref.		Ref.	
Male	1.105 (0.894-1.367)	0.037	1.268 (1.016-1.582)	0.036
Race				
White	Ref.			
Black	1.149 (0.920-1.435)	0.221		
Others^a^	1.127 (0.857-1.481)	0.393		
Marital status				
Married	Ref.			
Unmarried	1.039 (0.822-1.315)	0.748		
Others^b^	1.003 (0.809-1.243)	0.981		
Primary site				
Upper	Ref.			
Middle/Thoracic	0.888 (0.733-1.077)	0.229		
Lower	0.993 (0.809-1.219)	0.946		
Others^c^	1.216 (0.934-1.582)	0.146		
Grade				
Grade I-II	Ref.			
Grade III	1.077 (0.889-1.306)	0.448		
Tumor size (cm)				
≤ 6	Ref.			
>6	1.120 (0.918-1.366)	0.265		
T stage				
T1	Ref.		Ref.	
T2	0.990 (0.661-1.484)	0.963	1.111 (0.723-1.707)	0.632
T3	0.716 (0.579-0.885)	0.002	0.777 (0.604-0.999)	0.049
T4	1.526 (1.247-1.868)	<0.001	1.270 (1.000-1.614)	0.050
N stage				
N1	Ref.			
N2	1.138 (0.832-1.557)	0.417		
N3	1.253 (0.781-2.011)	0.350		
Radiotherapy				
No/Other	Ref.		Ref.	
Yes	0.654 (0.536-0.798)	<0.001	0.740 (0.603-0.907)	0.004
Chemotherapy				
No/Unknown	Ref.		Ref.	
Yes	0.302 (0.240-0.379)	<0.001	0.295 (0.234-0.373)	<0.001

HR, hazard ratio; 95% CI, 95% confidence interval; Others^a^, including Asian or Pacific Islander and American Indian/Alaska Native; Others^b^, including separated, divorced and widowed; Others^c^, including overlapping lesion of esophagus and not otherwise specified.

### Nomogram creation for the prediction of OS

**Figure 2:**
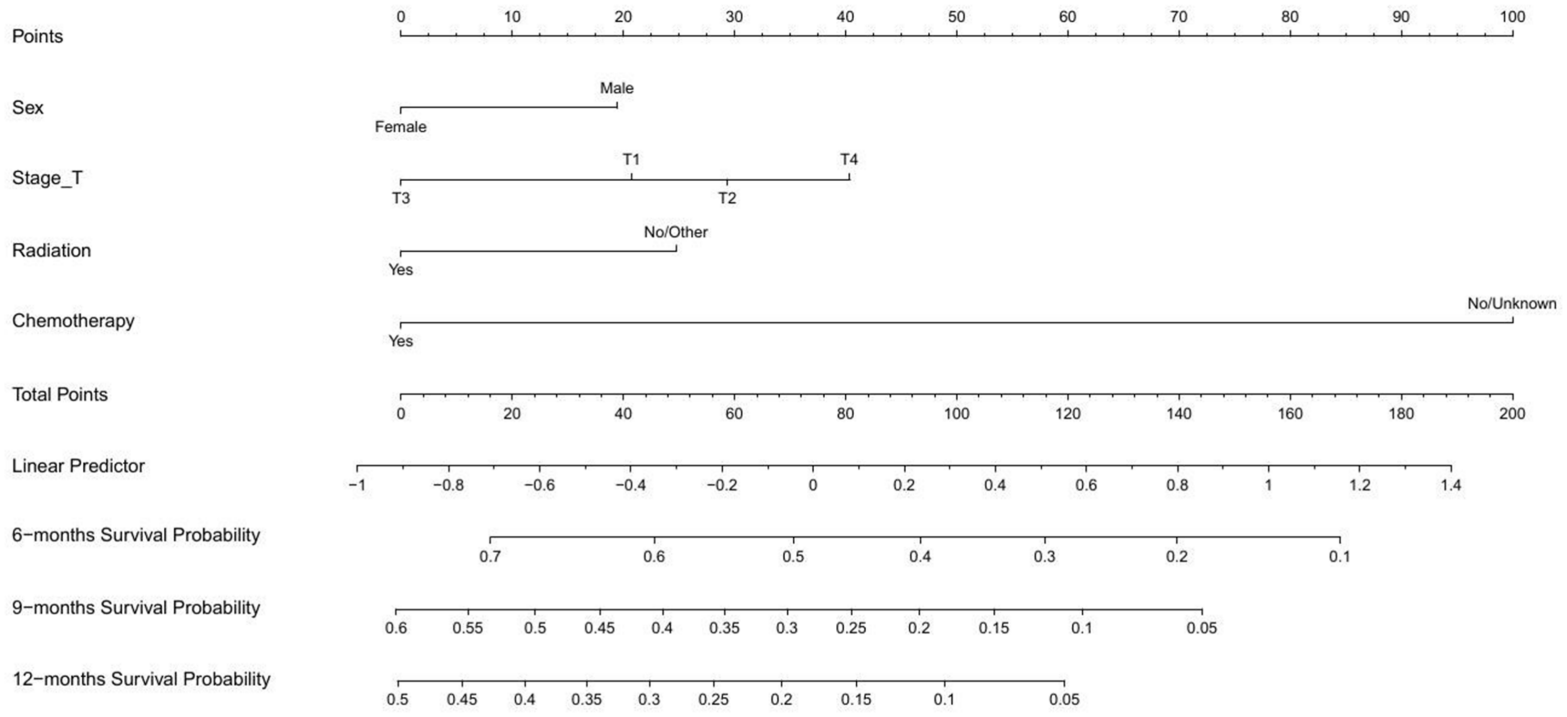
Nomogram for predicting 6-, 9-, and 12-month OS of patients with advanced ESCC.

As shown in **Figure 2**, a nomogram for predicting OS were established based on four variables screened above. This prognostic nomogram was a predictive tool for estimating the 6-, 9-month, or 1-year OS of ESCC patients with cT1-4N1-3M1 stage. It could be found that chemotherapy had the greatest impact on the OS prediction, followed by T stage from this nomogram. The line segment corresponding to each variable on the nomogram was marked with a scale, representing the range of values, and the length of the line segment reflected the relative contribution of the variable. The number of points for each variable was determined by drawing a line up to the point axis. The total score was obtained by adding the scores of all variables on the corresponding scale. A straight line was painted down from the total point to the straight line of 6-, 9-, and 12-month OS, and the intersection point was the 6-, 9-, and 12-month OS rate.

### Evaluation of nomogram

**Figure 3:**
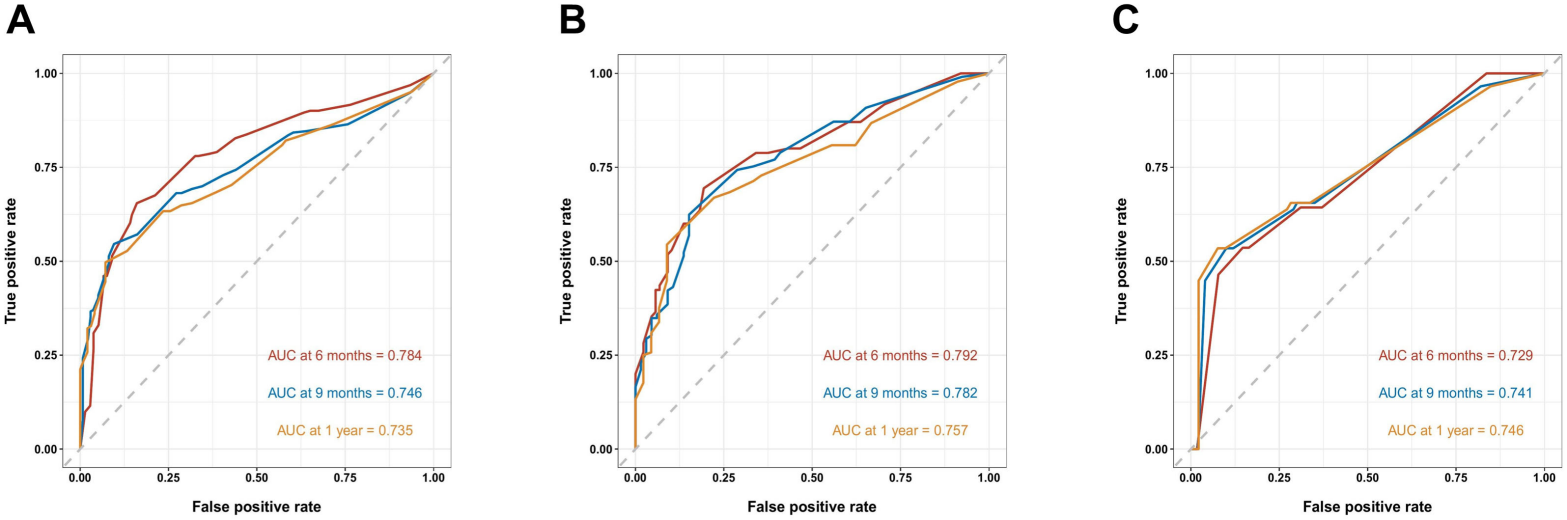
Receiver operating characteristic (ROC) curves of the nomogram predicting 6-, 9-, and 12-month OS in the training cohort **(A)**, the internal validation cohort **(B)** and the external training cohort **(C)**.

**Figure 4:**
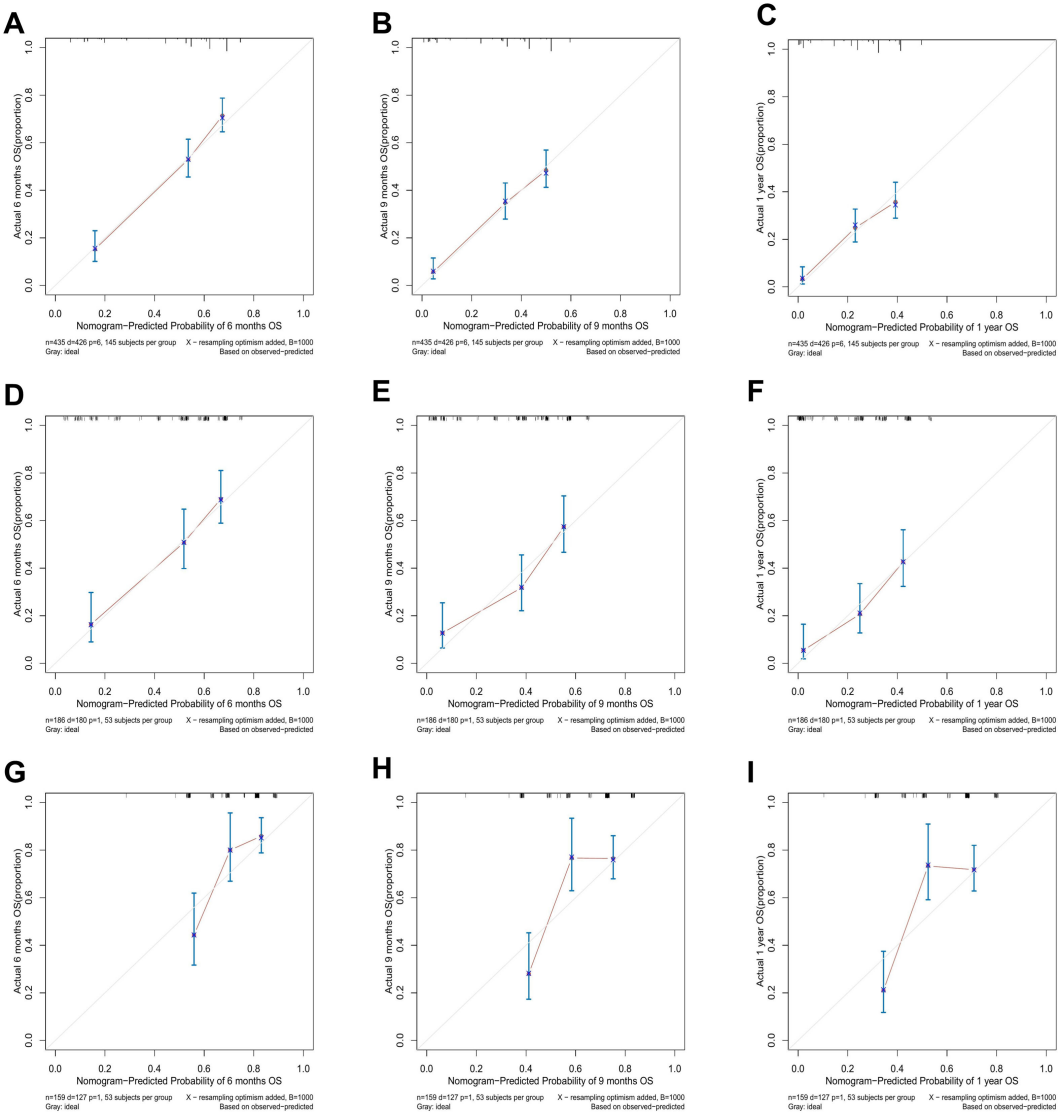
The calibration curves for predicting 6-, 9-, and 12-month OS in the training cohort **(A–C)**, the internal validation cohort **(D–F)**, and the external validation cohort **(G–I)**.

As shown in **Figure 3**, the prognostic nomogram in training cohort exhibited AUCs of 0.784, 0.746, and 0.735 for predicting 6-, 9-, and 12-month OS, respectively. In validation cohort, the nomogram still showed acceptable discrimination ability (AUCs in internal validation cohort: 0.792, 0.782, and 0.757; AUCs in external validation cohort: 0.729, 0.741, and 0.746). In **Figure 4**, the calibration curves were generated to suggest that the nomogram-predicted survival rates had good consistency with the observed survival rates in the actual population in both training and validation cohorts.

### Risk stratification based on nomogram

**Figure 5:**
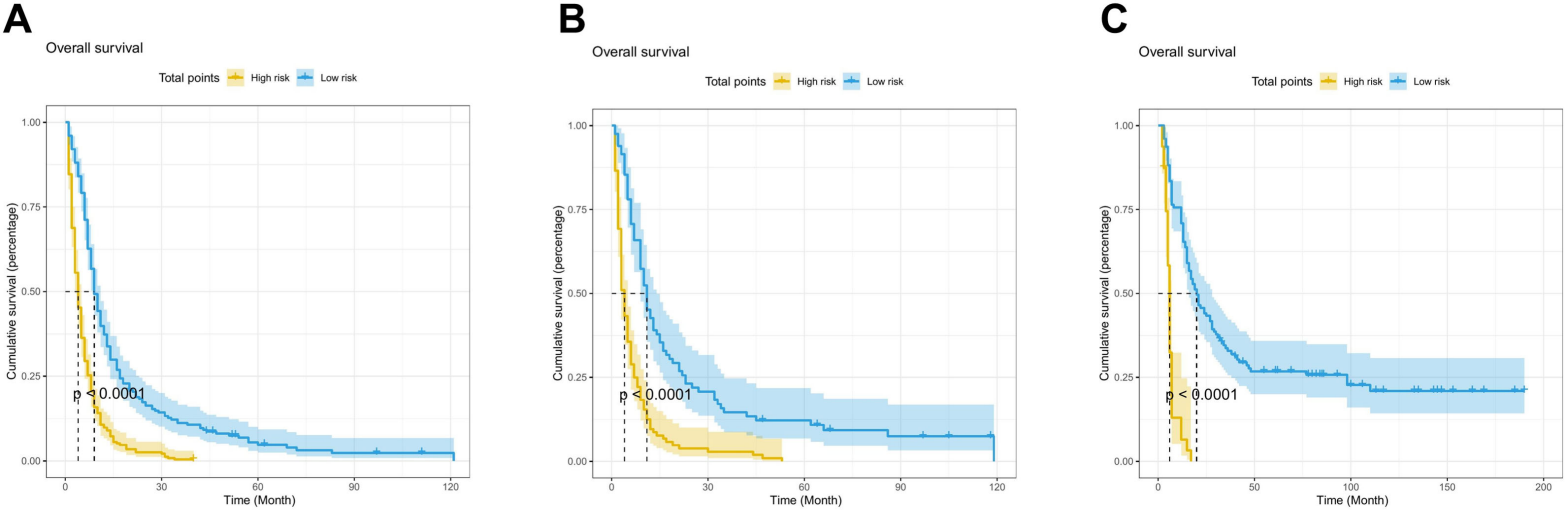
Kaplan-Meier curve of risk stratification for OS based on nomogram in the training cohort **(A)**, the internal validation cohort **(B)**, and the external validation cohort **(C)**.

After that, we calculated the predicted total points according to the established nomogram in three cohorts. The median risk score in the training cohort (59.78) was used as the cut-off value to subdivide patients into high-risk (risk score ≥ 59.78) and low-risk group (risk score < 59.78). As showed in **Figure 5**, the OS of the high-risk group was shorter than that of the low-risk group in three cohorts (all *P* < 0.0001). This revealed that nomogram could help us to accurately stratify risk in ESCC patients with cT1-4N1-3M1 stage.

## Discussion

Due to the heterogeneity of tumors and varying clinicopathological features, patients with EC can have different treatments and prognosis. In recent ten years, the prognosis of EC has gradually improved in many countries, and both the incidence and mortality have been decreasing. With the advancement of treatment technologies, EC has evolved from a single-disciplinary treatment model of radical surgical resection of tumors to an adjuvant therapy model of tumor cell removal, a gene and targeted immunotherapy model, and an individualized multidisciplinary integrated treatment model. However, many of the tumors will have progressed to advanced stages during the diagnosis, and the benefit rate of surgery alone in advanced patients was low (1, 2).

Previous studies have shown that many clinicopathological factors were closely related to the prognosis of ESCC, such as age, sex, marital status, primary site, grade, stage, lymphovascular invasion, treatment and so on (21–23). However, in our present study, some factors namely sex, T stage, radiotherapy, and chemotherapy affected survival, while the age, race, marital status, primary site, grade, tumor size, N stage had no significance on prognosis. Our study is the first to establish a nomogram for ESCC patients with cT1-4N1-3M1 stage. Moreover, our nomogram based on sex, T stage, radiotherapy, and chemotherapy is a novel prognostic model for this group of patients. In addition, our model was developed from SEER database and verified in the real world of the high incidence area of esophageal cancer in Chaoshan, China.

It has been generally believed that the depth of tumor invasion was closely related to the survival of EC (24). A significant interaction was found between the T stage and M stage when determining the OS of esophageal cancer. Deng et al. (25) proved that T1 stage predicted worse survival compared with T2 and T3 stage in metastatic esophageal cancer, while the survival rate is better when compared with T4. Our study had similar results, patients with T1 stage experienced significantly worse OS than those with T3 stage. However, there was no significant difference in OS between patients in stage T1 and those in T2 and T4.

In our study, sex was an independent risk factor affecting OS in ESCC patients with cT1-4N1-3M1 stage, as females had significantly higher survival rate than males. The majority of individuals in the three cohorts were male and had worse 1-year survival rates than female (training cohort: 22.0% vs. 30.4%; internal validation cohort: 26.3% vs. 28.6%; external validation cohort: 53.6% vs. 83.7%). Previous studies have also shown that the survival rate of women with EC was higher, which may be related to the poor lifestyle habits in male patients, such as smoking and drinking (26, 27), in addition to DNA damage and repair, sex hormones, sex hormone-related receptors, and tumor suppressor effects of the X chromosome (28). Accumulating evidences indicated that sex demonstrates different impacted on the outcome of immunotherapy in different tumor types. In melanoma and colorectal cancer, male patients showed more favorable survival outcomes (29–32), while in lung cancer, immunotherapy resulted in better survival benefits for female patients (33). In EC, it was well-established that the cases of male patients were significant more than female cases across various age, region, and tumor stage (34), suggesting that the biological basis associated with sex disparities play a significant role in this specific malignancy.

With the progress of medicine, there are many treatment methods for EC, including surgery, chemotherapy, radiation therapy, targeted therapy and immunotherapy, and all these methods can improve the survival rate of patients (15, 35, 36). Advanced ESCC has caused distant metastasis and obvious local invasion, which increases the difficulty of surgical resection and has a poor prognosis. Therefore, surgical resection might be not appropriate for advanced ESCC. Neoadjuvant chemoradiation was recommended for advanced ESCC patients. Chemotherapy medicines like carboplatin, paclitaxel, cisplatin, and fluorouracil were frequently selected in combination therapy with radiation, and the pathological complete response and overall survival rate were improved as compared with surgery alone (37, 38). Treatment goals of clinician were to improve the quality of life, control the complications, and prolong survival time through various treatment methods. A number of preclinical studies have shown that chemoradiotherapy can induce immunogenic cell death, enabling tumor cells to be recognized by the host immune system and triggering an immune response against the tumor (39, 40). In this study, we enrolled patients with advanced ESCC. Radiotherapy and chemotherapy are the main treatment modalities for these patients. Our model had shown that radiotherapy and chemotherapy are significantly associated with the clinical outcomes of patients with advanced ESCC (*P* < 0.05). Of course, when selecting treatment options, it is essential to make a comprehensive judgment based on the actual situation of each patient.

Based on the nomogram, we established a prognostic risk assessment that can classify patients into high- and low-risk groups. For high-risk patients, clinical physicians should adopt more aggressive and comprehensive interventions. In terms of treatment, combining radiotherapy, chemotherapy, and targeted therapy can enhance efficacy. Meanwhile, patients should increase the frequency of follow-ups to detect and address potential issues promptly. As for low-risk patients, current treatment can be maintained. Regular monitoring and follow-ups should be conducted to ensure stable conditions.

However, there were still unavoidable limitations in our study. First, this was a retrospective analysis based on a public database with certain confounding factors and unavoidable treatment bias that required prospective randomized clinical trials to provide a high-level evidence for clinical application. Second, many important clinical factors were missing from the SEER database, including smoking, alcohol consumption, genetic aspects, and specific drugs and radiotherapy regimens of chemotherapy and radiotherapy, which could not be ruled out the potential effect on prognosis. In addition, immunotherapy and targeted therapy have been shown to improve the prognosis in advanced ESCC patients, but this information was not recorded in the SEER database. Finally, our study population is highly heterogeneous, which may have a certain impact on the accuracy and reliability of the study results. Although we conducted external validation to demonstrate the predictive value of our model, there are some differences between the local center data and the training cohort data, which may have an impact on the feasibility of evaluating the model’s performance. In future research, we would consider further in-depth analysis of the prognostic characteristics of different subtypes and optimize the model construction and validation process for specific subtypes. In addition, the sample size was not large enough, which may introduce bias. Therefore, data from different institutions should be used to further validate and optimize our findings, ensuring that they can play a positive role in a broader patient population.

## Conclusion

In the present study, we obtained clinicopathological data of patients from the SEER database, and constructed and validated a nomogram for prognosis prediction of ESCC patients with cT1-4N1-3M1 stage. The reliability and accuracy of the model were also observed as satisfactory.

## Data Availability

The raw data supporting the conclusions of this article will be made available by the authors, without undue reservation.

## References

[1] Bray F Laversanne M Sung H Ferlay J Siegel RL Soerjomataram I. Global cancer statistics 2022: GLOBOCAN estimates of incidence and mortality worldwide for 36 cancers in 185 countries. CA Cancer J Clin. (2024) 74:229–63. doi: 10.3322/caac.21834 38572751

[2] Abnet CC Arnold M Wei WQ. Epidemiology of esophageal squamous cell carcinoma. Gastroenterology. (2018) 154:360–73. doi: 10.1053/j.gastro.2017.08.023 PMC5836473 28823862

[3] Rice TW. Superficial oesophageal carcinoma: is there a need for three-field lymphadenectomy? Lancet. (1999) 354:792–4. doi: 10.1016/S0140-6736(99)80005-1 10485718

[4] Prenzel KL Bollschweiler E Schroder W Monig SP Drebber U Vallboehmer D. Prognostic relevance of skip metastases in esophageal cancer. Ann Thorac Surg. (2010) 90:1662–7. doi: 10.1016/j.athoracsur.2010.07.008 20971285

[5] Hsu WH Hsu PK Hsieh CC Huang CS Wu YC. The metastatic lymph node number and ratio are independent prognostic factors in esophageal cancer. J Gastrointest Surg. (2009) 13:1913–20. doi: 10.1007/s11605-009-0982-8 19672664

[6] Ichida H Imamura H Yoshimoto J Sugo H Kajiyama Y Tsurumaru M. Pattern of postoperative recurrence and hepatic and/or pulmonary resection for liver and/or lung metastases from esophageal carcinoma. World J Surg. (2013) 37:398–407. doi: 10.1007/s00268-012-1830-7 23142988

[7] Robb WB Messager M Dahan L Mornex F Maillard E D’journo XB. Patterns of recurrence in early-stage oesophageal cancer after chemoradiotherapy and surgery compared with surgery alone. Br J Surg. (2016) 103:117–25. doi: 10.1002/bjs.9959 26511668

[8] Shiozaki H Sudo K Xiao L Wadhwa R Elimova E Hofstetter WL. Distribution and timing of distant metastasis after local therapy in a large cohort of patients with esophageal and esophagogastric junction cancer. Oncology. (2014) 86:336–9. doi: 10.1159/000360703 PMC4105702 24925190

[9] Kato K Sun JM Shah MA Enzinger PC Adenis A Doi T. Pembrolizumab plus chemotherapy versus chemotherapy as first-line therapy in patients with advanced esophageal cancer: The phase 3 KEYNOTE-590 study. Ann Oncology. (2020) 31:S1142–S215. doi: 10.1016/j.annonc.2020.08.2298

[10] Shah MA Sun JM Shen L Kato K. First-line pembrolizumab (pembro) plus chemotherapy (chemo) for advanced esophageal cancer: 5-year outcomes from the phase 3 KEYNOTE-590 study. J Clin Oncol. (2024) 42:250. doi: 10.1200/JCO.2024.42.3_suppl.250 37883737

[11] Wu SG Zhang WW Sun JY Li FY Lin Q He ZY. Patterns of distant metastasis between histological types in esophageal cancer. Front Oncol. (2018) 8:302. doi: 10.3389/fonc.2018.00302 30135855 PMC6092597

[12] Yao Y Liu Z Li Q Cao B Wang M. Successful immune checkpoint inhibitor-based rechallenge in a patient with advanced esophageal squamous cell cancer: A case report. Thoracic Cancer. (2022) 13:497–501. doi: 10.1111/1759-7714.14279 35014762 PMC8807265

[13] Ng HY Ko JMY Lam KO Kwong DLW Lo AWI Wong IYH. Circulating tumor DNA dynamics as prognostic markers in locally advanced and metastatic esophageal squamous cell carcinoma. JAMA Surg. (2023) 158:1141–50. doi: 10.1001/jamasurg.2023.4395 PMC10512170 37728901

[14] Yang W Han Y Zhao X Duan L Zhou W Wang X. Advances in prognostic biomarkers for esophageal cancer. Expert Rev Mol Diagn. (2019) 19:109–19. doi: 10.1080/14737159.2019.1563485 30582379

[15] Han LQ Cui TT Xiao NJ Li W. Prognostic analysis and treatment utilization of different treatment strategies in elderly esophageal cancer patients with distant metastases: a SEER database analysis. J Cancer Res Clin Oncol. (2023) 149:15413–23. doi: 10.1007/s00432-023-05260-6 PMC11796667 37644234

[16] Huang Y Yu X Li W Li Y Yang J Hu Z. Development and validation of a nomogram for predicting late-onset sepsis in preterm infants on the basis of thyroid function and other risk factors: Mixed retrospective and prospective cohort study. J Advanced Res. (2020) 24:43–51. doi: 10.1016/j.jare.2020.02.005 PMC7063096 32181015

[17] Makkouk A Sundaram V Chester C Chang S Colevas AD Sunwoo JB. Characterizing CD137 upregulation on NK cells in patients receiving monoclonal antibody therapy. Ann Oncology. (2017) 28:415–20. doi: 10.1093/annonc/mdw570 PMC6246233 27831501

[18] Yang N Xu L Wang Q Chen F Zhou Y. Construction and validation of a prognostic nomogram for anal squamous cell carcinoma. Cancer Med. (2022) 11:392–405. doi: 10.1002/cam4.4458 34850581 PMC8729044

[19] Zhang X Chang L Zhu Y Mao Y Zhang T Zhang Q. Establishment and validation of nomograms to predict survival probability of advanced Malignant pleural mesothelioma based on the SEER database and a Chinese medical institution. Front Endocrinology. (2023) 14:1139222. doi: 10.3389/fendo.2023.1139222 PMC10140559 37124752

[20] Li J Liu Y Yan Z Wan X Xia Y Wang K. A nomogram predicting pulmonary metastasis of hepatocellular carcinoma following partial hepatectomy. Br J Cancer. (2014) 110:1110–7. doi: 10.1038/bjc.2014.19 PMC3950869 24481404

[21] Chen H Wu J Guo W Yang L Lu L Lin Y. Clinical models to predict lymph nodes metastasis and distant metastasis in newly diagnosed early esophageal cancer patients: A population-based study. Cancer Medicine. (2022) 12:5275–92. doi: 10.1002/cam4.5334 PMC10028124 36205033

[22] Kang M Wang Y Yang M Wang X Zhu L Zhang M. Prognostic nomogram and risk factors for predicting survival in patients with pT2N0M0 esophageal squamous carcinoma. Sci Reports. (2023) 13:4931–39. doi: 10.1038/s41598-023-32171-w PMC10040408 36967413

[23] Wang Y Zhu L Xia W Wu L Wang F. The impact of adjuvant therapies on patient survival and the recurrence patterns for resected stage IIa–IVa lower thoracic oesophageal squamous cell carcinoma. World J Surg Oncology. (2018) 16:216–4. doi: 10.1186/s12957-018-1516-1 PMC6223077 30404621

[24] Nentwich MF Von Loga K Reeh M Uzunoglu FG Marx A Izbicki JR. Depth of submucosal tumor infiltration and its relevance in lymphatic metastasis formation for T1b squamous cell and adenocarcinomas of the esophagus. J Gastrointest Surg. (2014) 18:242–9; discussion 49. doi: 10.1007/s11605-013-2367-2 24091912

[25] Deng J Chu X Ren Z Wang B. Relationship between T stage and survival in distantly metastatic esophageal cancer: A STROBE-compliant study. Med (Baltimore). (2020) 99:e20064. doi: 10.1097/MD.0000000000020064 PMC7220676 32384472

[26] Kauppila JH Wahlin K Lagergren P Lagergren J. Sex differences in the prognosis after surgery for esophageal squamous cell carcinoma and adenocarcinoma. Int J Cancer. (2019) 144:1284–91. doi: 10.1002/ijc.31840 30168595

[27] Shen W-B Gao H-M Zhu S-C Li Y-M Li S-G Xu J-R. Analysis of the causes of failure after radical surgery in patients with PT3N0M0 thoracic esophageal squamous cell carcinoma and consideration of postoperative radiotherapy. World J Surg Oncology. (2017) 15:192–98. doi: 10.1186/s12957-017-1259-4 PMC5657067 29070049

[28] Rubin JB. The spectrum of sex differences in cancer. Trends Cancer. (2022) 8:303–15. doi: 10.1016/j.trecan.2022.01.013 PMC8930612 35190302

[29] Hodi FS Chesney J Pavlick AC Robert C Grossmann KF Mcdermott DF. Combined nivolumab and ipilimumab versus ipilimumab alone in patients with advanced melanoma: 2-year overall survival outcomes in a multicentre, randomised, controlled, phase 2 trial. Lancet Oncol. (2016) 17:1558–68. doi: 10.1016/S1470-2045(16)30366-7 PMC5630525 27622997

[30] Ribas A Kefford R Marshall MA Punt CJ Haanen JB Marmol M. Phase III randomized clinical trial comparing tremelimumab with standard-of-care chemotherapy in patients with advanced melanoma. J Clin Oncol. (2013) 31:616–22. doi: 10.1200/JCO.2012.44.6112 PMC4878048 23295794

[31] Schachter J Ribas A Long GV Arance A Grob JJ Mortier L. Pembrolizumab versus ipilimumab for advanced melanoma: final overall survival results of a multicentre, randomised, open-label phase 3 study (KEYNOTE-006). Lancet. (2017) 390:1853–62. doi: 10.1016/S0140-6736(17)31601-X 28822576

[32] White A Ironmonger L Steele RJC Ormiston-Smith N Crawford C Seims A. A review of sex-related differences in colorectal cancer incidence, screening uptake, routes to diagnosis, cancer stage and survival in the UK. BMC Cancer. (2018) 18:906–16. doi: 10.1186/s12885-018-4786-7 PMC6149054 30236083

[33] Conforti F Pala L Bagnardi V Viale G De Pas T Pagan E. Sex-based heterogeneity in response to lung cancer immunotherapy: A systematic review and meta-analysis. JNCI: J Natl Cancer Institute. (2019) 111:772–81. doi: 10.1093/jnci/djz094 PMC6695312 31106827

[34] Siegel RL Miller KD Jemal A. Cancer statistics, 2019. CA Cancer J Clin. (2019) 69:7–34. doi: 10.3322/caac.21551 30620402

[35] Cowzer D Wu AJ Sihag S Walch HS Park BJ Jones DR. Durvalumab and PET-directed chemoradiation in locally advanced esophageal adenocarcinoma: A phase ib/II study. Ann Surg. (2023) 278:e511–e18. doi: 10.1097/SLA.0000000000005818 PMC11065504 36762546

[36] Manish A Shah M Erin B Alarcon-Rozas A. Immunotherapy and targeted therapy for advanced gastroesophageal cancer: ASCO guideline. J Clin Oncol. (2023) 41):1470–91. doi: 10.1200/JCO.22 36603169

[37] Tepper J Krasna MJ Niedzwiecki D Hollis D Reed CE Goldberg R. Phase III trial of trimodality therapy with cisplatin, fluorouracil, radiotherapy, and surgery compared with surgery alone for esophageal cancer: CALGB 9781. J Clin Oncol. (2008) 26:1086–92. doi: 10.1200/JCO.2007.12.9593 PMC5126644 18309943

[38] Van H Hulshof M Steyerberg E Van Lanschot J Henegouwen M Wijnhoven B. Preoperative chemoradiotherapy for esophageal or junctional cancer. New Engl J Med. (2012) 366:2074–84. doi: 10.1056/NEJMoa1112088 22646630

[39] Bracci L Schiavoni G Sistigu A Belardelli F. Immune-based mechanisms of cytotoxic chemotherapy: implications for the design of novel and rationale-based combined treatments against cancer. Cell Death Differ. (2014) 21:15–25. doi: 10.1038/cdd.2013.67 23787994 PMC3857622

[40] Gameiro SR Jammeh ML Hodge JW Wattenberg MM. Radiation-induced immunogenic modulation of tumor enhances antigen processing and calreticulin exposure, resulting in enhanced T-cell killing. Oncotarget. (2014) 5:403–16. doi: 10.18632/oncotarget.1719 PMC3964216 24480782

